# Mitogenomic analysis of Thai Sunda pangolins reveals regional phylogeography and informs conservation management

**DOI:** 10.1038/s41598-025-97182-1

**Published:** 2025-04-23

**Authors:** Nattapong Banterng, Kyle Ewart, Frankie Thomas Sitam, Rob Ogden

**Affiliations:** 1 Royal (Dick) School of Veterinary Studies and the Roslin Institute, University of Edinburgh, Easter Bush Campus, Edinburgh, EH25 9RG UK; 2 Department of National Parks, Wildlife and Plant Conservation, Bangkok, Thailand; 3 School of Life and Environmental Sciences, University of Sydney, Sydney, NSW 2050 Australia; 4 TRACE Wildlife Forensics Network, Edinburgh, EH12 6LE UK; 5 Department of Wildlife and National Parks (DWNP/PERHILITAN), National Wildlife Forensic Laboratory (NWFL), Kuala Lumpur, Malaysia

**Keywords:** Mitochondrial DNA, Conservation genetics, Phylogeography, Endangered species, Traceability, Molecular ecology, Population genetics

## Abstract

Pangolins are considered the most trafficked mammals in the world with all eight species listed on CITES Appendix I. Despite this pervasive threat to their survival, there remains a limited understanding of genetic diversity and connectivity among populations of Asian pangolin species, hampering effective conservation management. We analysed mitogenome sequences of the Sunda pangolin (*Manis javanica*) from across their Southeast Asia continental distribution, as well as Borneo. Phylogenetic reconstruction revealed six lineages, with clear separation north and south of the Kangar-Pattani biogeographic line in southern Thailand, revealing clear differentiation between Sundaland and Indochinese Sunda pangolin lineages. Further divergence across an east–west divide was observed in central and northern Thailand, extending northwards towards China. Our results provide new insights into the evolutionary relationships among Sunda pangolin populations in Southeast Asia, building on other recent research in this field and helping to establish the species’ baseline phylogeography. These inferences will aid conservation planning and support the genetic traceability of the illegal pangolin trade.

## Introduction

Pangolins are recognised as “the most heavily trafficked wild mammal in the world”^1,2^; between 2000–2019, it is estimated that around one million pangolins were trafficked globally^3^. According to the International Union for Conservation of Nature Red List of Threatened Species^4^, all eight pangolin species in Africa and Asia are now threatened with extinction, with three of the Asian pangolins (Chinese pangolin (*Manis pentadactyla*), Philippine pangolin (*Manis culionensis*) and Sunda pangolin (*Manis javanica*)) considered to be Critically Endangered. Moreover, all pangolin species have been listed in CITES Appendix I since January 2017, which means international trade in pangolins is prohibited^5^. The Sunda pangolin is one of four Asian pangolin species distributed across Southeast Asia and southern China and living in varied habitats from primary forest to urban areas^6,7^ and the most trafficking pangolin in the pre-2000 period^3^. The illegal wildlife trade is driven by a demand for pangolin scales for use in traditional medicine and pangolin meat, which is considered a delicacy^8^ and Southeast Asia plays an important role in the pangolin trade as a source, hub, and destination^9^. As a result of increasing demand in Asia and the consequent decline of Asian pangolins, African pangolins are also now targeted for illegal trade into the Asian market^10–13^; however, the pressure on Asian pangolins persists, they are still hunted and trafficked internationally^14^ and the number of individual pangolins in illegal trade is still increasing^3,15^.

Over the last decade, pangolins have received considerable scientific attention, with researchers primarily focusing on pangolin conservation, but also on pangolin biology^16^. Despite this rise in interest, significant gaps in our knowledge of pangolins remains^16^, with the recent finding of a possible fifth species of Asian pangolin based solely on seized scale samples^17^ emphasizing our lack of fundamental zoological knowledge. Within widely distributed species there is relatively little understanding of geographic variation and population structure.

Mitochondrial DNA sequencing is well-recognised as a primary source of information for reconstructing phylogenetic relationships among species^18,19^, to identify conservation units within species and to aid in the forensic identification of wildlife evidence. For pangolins, it has been shown that mitochondrial DNA genes can be used for species assignment of scales or degraded samples^11,20–26^. The ability to identify the geographic origin of seizures is more limited although seizures of white-bellied pangolin (*Phataginus tricuspis*) have been identified to one of six geographic lineages throughout the species range via mtDNA sequencing^24,25^.

The Sunda pangolin is one of the most studied pangolin species^16^. A recent mtDNA genome phylogenetic study of the Sunda pangolin in Malaysia showed a clear distinction between samples from northern Borneo and those from west/south Borneo, Peninsular Malaysia and mainland Southeast Asia population^27^ and consistent with the nuclear genome study that distinguished this species into two populations with five subpopulations^28^, demonstrating the potential for regional geographic assignment. However, although the species distribution is arguably centered around Thailand, there is limited information on phylogeographic structure in this region. This is required both to inform conservation management and enable traceability of pangolin seizures for law enforcement in Thailand and the wider region. This study aims to characterize the phylogeographic structure and biogeography of the Sunda pangolin in Thailand to enhance our knowledge of the species biology, and to support tools for conservation genetic management and wildlife DNA forensics.

## Materials and methods

### Ethics statement

This study, in compliance with the Animal Research: Reporting of In Vivo Experiments (ARRIVE) guidelines for animal research and ethical approval to collect the samples was received from the University of Edinburgh Animal Welfare and Ethical Review Board (AWERB) (No. OS2-22). Permission to collect samples from Sunda pangolin, both dead and alive, from inside and outside-protected areas was granted under the Wildlife Protection and Preservation Act of 2562 B.E. from the Department of National Parks Wildlife and Plant Conservation, Thailand (Permit No.0909.204/9311), and the procedure in this study involve with animals were carried out in accordance to the Wildlife Protection and Preservation Act of 2562 B.E. and relevant regulations. The Sunda pangolin samples (blood and tissues) in this project were imported to the University of Edinburgh, UK, from Thailand under CITES export permit No. 22TH0902.2/521 and 23TH0902.2/2 (Thailand) and the CITES import permit No. 616968/01-03 and 624109/01 (UK).

### Collection and DNA extraction

Sunda pangolin blood (*n* = *25*) and tissue (*n* = *6*) samples were collected opportunistically across Thailand (Sup. Figure S1); the samples were either from rescued or confiscated pangolins of known geographic origin. The geographical origin of confiscated specimens was based on enforcement records, as the animals were seized directly from local hunters who were apprehended within specific protected areas, and who confirmed the collection locations during their arrest. Given the nature of these enforcement operations, it is extremely unlikely that any of these samples derived from long-distance trafficking operations. The blood samples were collected in EDTA tubes and the tissue samples were collected in plastic tubes and stored at − 20 °C before analysis. DNA was extracted using the PureLink™ Genomic DNA Mini Kit (Cat. No. K182001) following the manufacturer’s protocol. Existing Sunda pangolin sequence data were downloaded from NCBI GenBank (Sup. Table S1).

### Genome sequencing and assembly

The 31 DNA samples were subject to whole genome sequencing using the Illumina NovaSeq platform (provided by Azenta Life Sciences). Paired-end 150 bp reads were generated to target an average sequencing depth of coverage of 10X across the genome. All paired-end reads were trimmed to remove low quality bases and adapter sequences using TrimGalore 0.6.6, with trimming parameters set to remove bases with Phred quality scores < 30 and to discard reads shorter than 35 bp after trimming. The trimmed reads were then mapped against a reference mitogenome from NCBI (accession number NC026781)^29^ with the BWA-MEM algorithm 0.7.17 and converted from SAM to BAM files with Samtools 1.9. BAM files were visualised and the mitogenome extracted in Geneious Prime 2022.2.2. Paired-end read data from NCBI (SRR9018664-5, SRR9018633, SRR25256520 and SRR25256582) were processed using the same method.

### Data analysis

In addition to the 31 mitogenomes generated in this study, a further 27 sequences of Sunda pangolin were obtained from the NCBI database with known origins from Peninsular Malaysia (*n* = 14), Borneo (*n* = 8)^27^, China (SRR9018664-5^28^; MG196309^30^ *n* = 3), Myanmar (SRR9018633^28^; *n* = 1) and the only previous sequence from the south of Thailand (MG196302^30^; *n* = 1). One Chinese pangolin (*Manis pentadactyla*) sequence (MG196305)^30^ was used as the outgroup. Additionally, two haplotypes from the recently proposed new Southeast Asian pangolin species, *Manis mysteria*^17^, were included to assess their evolutionary relationship with the Thai Sunda pangolin samples. These 61 mitogenomes were aligned using MAFFT v7.490^31^ following the L-INS-i method and ambiguously aligned positions removed with Gblocks v.0.91b^32,33^ with less stringent selection (allowing smaller final blocks, gap positions and fewer strict flanking positions).

We used PartitionFinder2^34^ in PhyloSuite 1.2.3^35,36^ to determine the best-fit partitioning scheme and substitution model. We defined the 23 transfer RNAs (tRNAs), ribosomal RNAs (rRNAs), d-loop and the 13 protein-coding genes as initial data blocks, and tested partitioning schemes based on the ‘greedy’ algorithm with branch lengths estimated as ‘linked’ with ‘all’ models to search for the optimal schemes. The corrected Akaike Information Criterion (AICc) was used to select the most optimal scheme. While other model selection criteria such as BIC and AIC are available, we selected AICc as it is particularly suitable for smaller sample sizes and provides a good balance between model fit and complexity^37^.

Phylogenetic reconstruction was performed using both a Maximum Likelihood approach in IQ-TREE v1.6.12 (1000 bootstraps)^38^, and a Bayesian approach performed with MrBayes v3.2.7a^39^ (2 parallel runs, 2 M generations, 25% burn-in). Convergence was checked using Tracer v1.7^40^ with two independent runs. The Effective Sample Sizes (ESS) values of 563.5 and 398.2 were well above the recommended threshold of 200^41^. Both methods used the best-fitting models for 14 partitions selected from PartitionFinder2. The resulting trees were visualised using FigTree v1.4.4^42^. Haplotype designations and networks were generated in PopART v.1.7^43^ using the Templeton-Crandall-Sing (TCS) algorithm using the sequence alignment after removing the hypervariable mtDNA control region and haplotype statistics using DnaSP v.6^44^.

To examine geographic structuring within the data, we notionally divided a subset of the study samples into five groups according to sampling locality. Two groups were based on established forest complexes in Thailand: western forest complex (W. Forest, *n* = 5), Khao Yai forest complex (KY forest, *n* = 11), and the remaining three groups were defined by geographic regions: mid-southern Thailand (Mid-south, *n* = 7), northern Borneo (N. Borneo, *n* = 7) and the Thailand-Malaysia border region (TH-MY border, *n* = 20) comprising samples from far south Thailand and peninsula Malaysia (Sup. Table S1). Occasional samples outside these areas were excluded from this analysis. Analysis of molecular variance (AMOVA) and *F*_ST_ statistics were calculated in Arlequin 3.5.2.2^45^, using the same sequences as the haplotype network and phylogenetic tree analysis (2,000 AMOVA permutations; 200 pairwise *F*_ST_ permutations, α = 0.05).

## Results

### Phylogenetic reconstruction

**Fig. 1:**
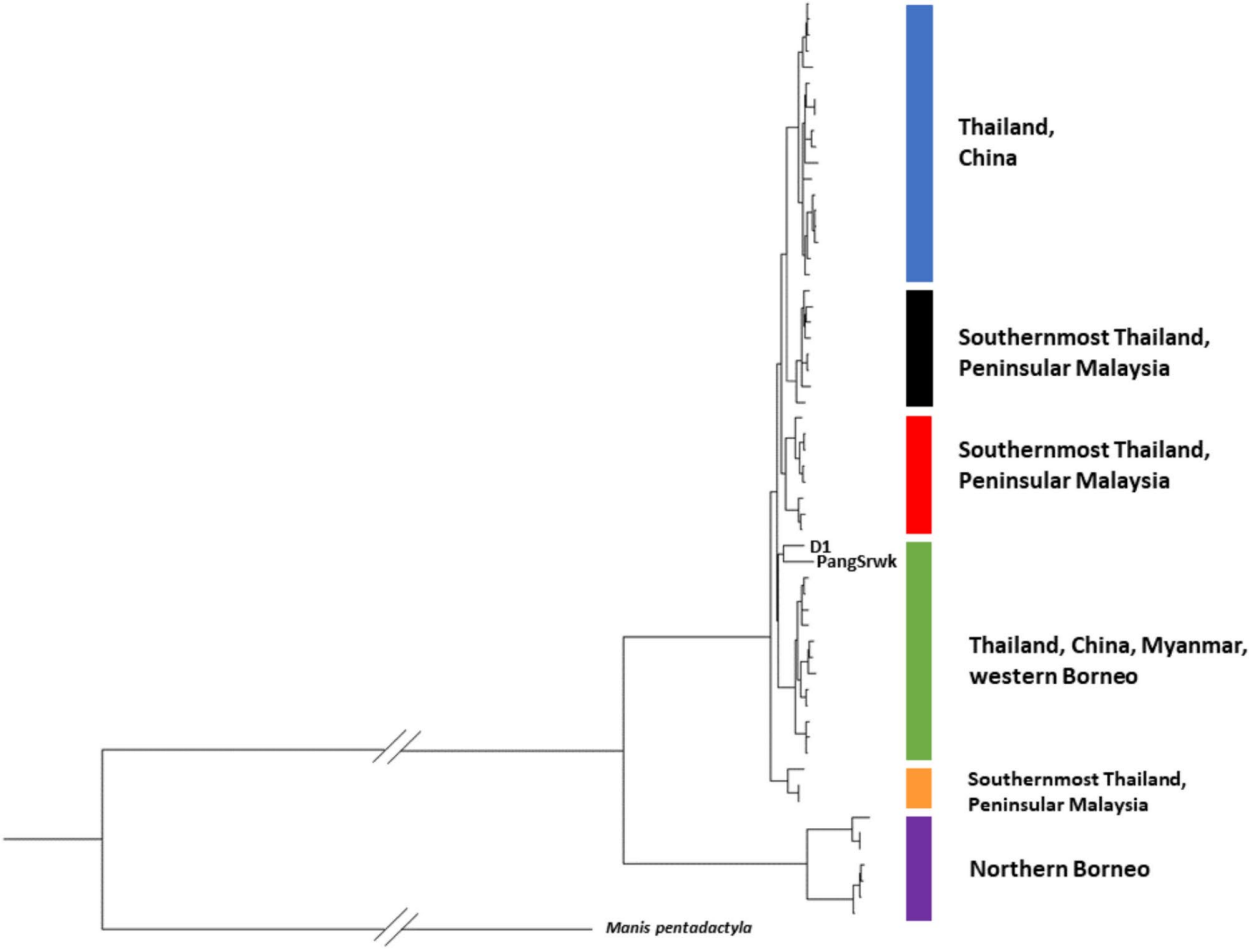
Bayesian phylogenetic reconstruction of the Sunda pangolin based on mitogenome sequences (16,000 bp). Color-coding refers to clades with Bayesian posterior probabilities ≥ 0.99. D1 sample was from the Southernmost Thailand and PangSrwk was from western Borneo.

A total of 52 novel Sunda pangolin mitogenome haplotypes were observed from the 31 samples sequenced in this study and the 27 retrieved from GenBank. A haplotype nomenclature system for Sunda pangolin mitogenomes including previously published sequences has been proposed (Sup. Table S1). Partition finder found highest support for fourteen blocks with differing substitution models (Sup. Table S4). The maximum likelihood and Bayesian trees show consistent clustering, with a principal division between northern Borneo and all other regions; primarily continental Southeast Asia (Peninsular Malaysia, Thailand, China, and Myanmar), but also a single sample from western Borneo (‘PangSrwk’, Fig. 1). Note, only one mitogenome was available from the western/southern Borneo lineage characterised in Sitam et al., (2023) (i.e., ‘PangSrwk’); the other two Kalimantan samples analysed in this study have only partial mtDNA gene regions available. Within continental Southeast Asia, five distinct clades with high support (≥ 0.99 Bayesian posterior probability) can be identified (Blue, Black, Red, Green and Orange; Fig. 1).

The Peninsular Malaysia and southernmost Thailand samples grouped predominantly into three clades (black, red and orange; Fig. 1), with overlapping sympatric genetic distributions. Interestingly, these Thai-Malay border clades did not form a phylogeographically cohesive group with respect to the blue and green clades observed further north in Thailand, China, and Myanmar, which exhibited a much wider geographic distribution (> 1000 km north–south; Fig. 3). One sample from southernmost Thailand (D1) formed an identifiable cluster with one from western Borneo (PangSrwk), with relatively longer branch lengths (Fig. 1), but with bootstrap support in the maximum likelihood tree (45%) and the Bayesian posterior probabilities (0.5) were both low (Sup. Figure S2 and S3), resulting in them being indistinguishable from the green clade under both phylogenetic analyses.

The two mitogenomes from the recently proposed new Asian pangolin species, *Manis mysteria*^17^, form a clade this is sister to all of the known Sunda pangolins included in this study (Sup. Figure S5). This *Manis mysteria* clade is genetically more similar to the Sunda pangolin than to the Chinese pangolin, *Manis pentadactyla*.

### Haplotype network

**Fig. 2:**
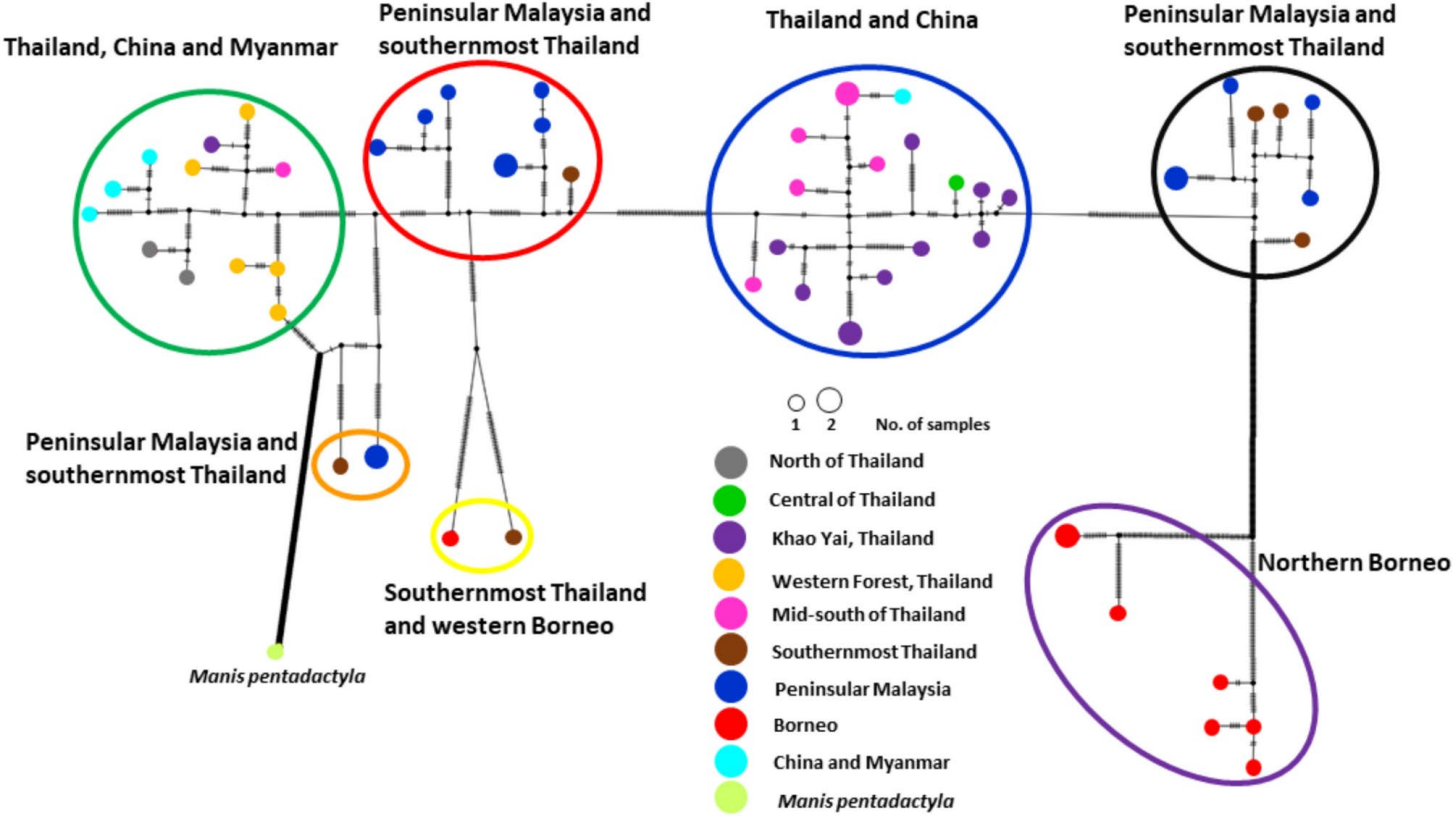
Haplotype network based on mitogenome (15,408 bp) of the samples from Thailand, Peninsular Malaysia, Borneo, China, and Myanmar. The diameter of haplotype circle reflects the total number of samples carrying this haplotype, the small black circle represents the missing haplotype and hatch-marks across lines represent mutational steps between haplotypes. Ellipse colours match those used in the phylogenetic tree (Fig. 1).

The haplotype network suggests seven genetic clusters, with the two samples from western Borneo and southernmost Thailand more clearly distinguished than under phylogenetic analysis (Fig. 2, yellow circle). The remaining six clusters correspond to the clades observed in the phylogenetic trees, although the network reveals considerable haplotype diversity within many of these, notably the red and orange clusters from the Thai-Malay border and purple cluster from northern Borneo.

**Fig. 3:**
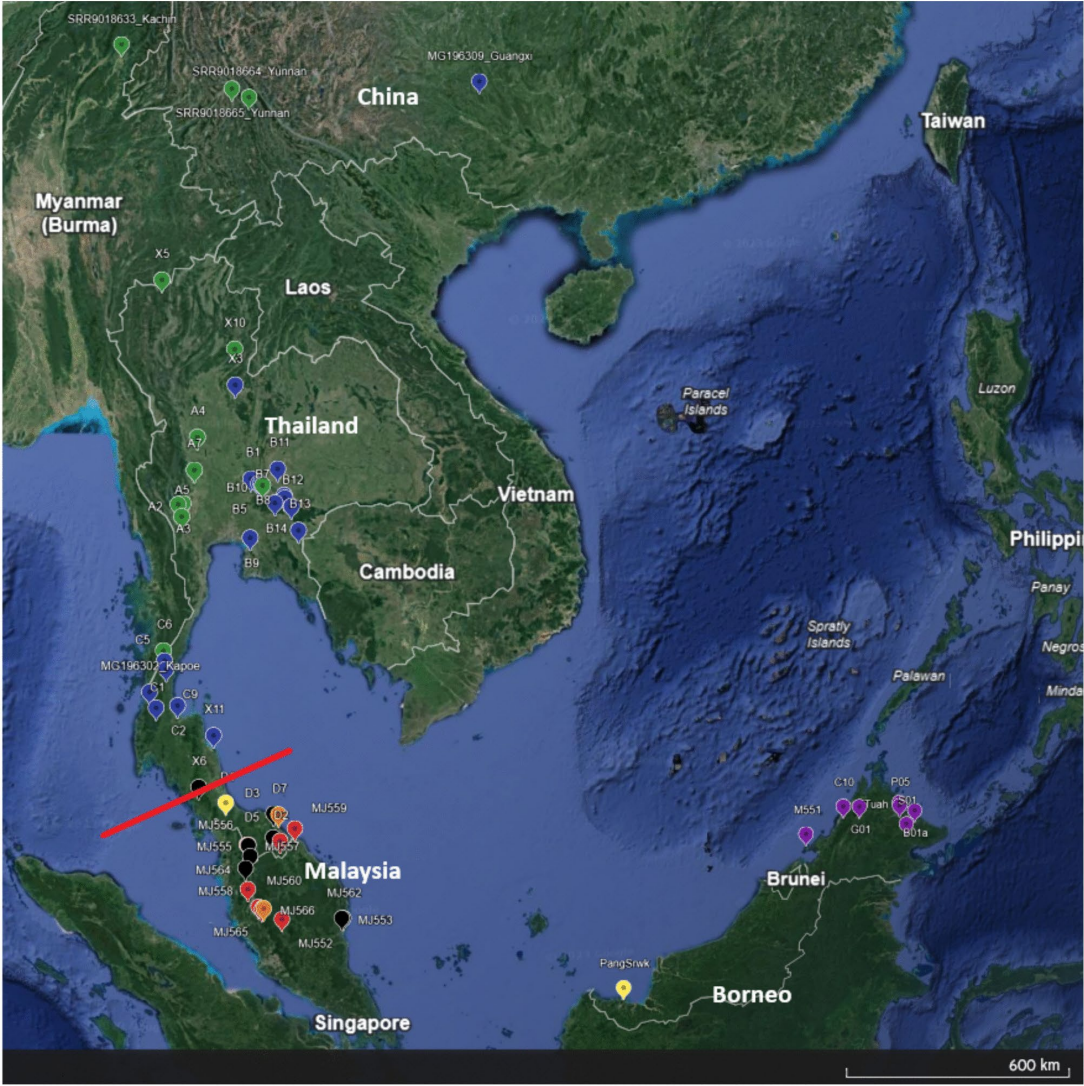
Distribution map of Sunda pangolin samples/sequence data used in this study, from Thailand, Peninsular Malaysia, Bornean Malaysia, China, and Myanmar. Colours represent clusters according to the haplotype network and the phylogenetic tree, and the red line represents the Kangar-Pattani Line (KPL). The map created using Google Earth, v. 10.69.0.1 (https://earth.google.com/).

In terms of geographic distribution, pangolin samples from the mid-south area and Khao Yai forest complex were grouped together mostly with one sample from central area and one sample from Guanxi, China (Blue clade). Haplotypes from the western forest complex group together along with one from mid-south of Thailand and other from southern China and northern Myanmar (green clade) (Figs. 2 and 3). All haplotypes observed in Peninsular Malaysia and southernmost Thailand were placed within the red, black, and orange clusters, but these showed significant differentiation from one another in terms of mutation steps.

Analysis of haplotype diversity revealed high genetic variation across most groups. When analysed by phylogenetic clades, six of the seven groups showed high haplotype diversity (Hd > 0.95), only the orange clade showed relatively lower diversity (Hd = 0.67) (Sup. Table S2). Geographic analysis showed similarly high diversity across regions, with the Thai-Malaysia border region containing 17 haplotypes among 20 samples (Hd = 0.98), and maximum diversity (Hd = 1.0) in both mid-south and western regions (Sup. Table S3).

### Geographic structure

**Fig. 4:**
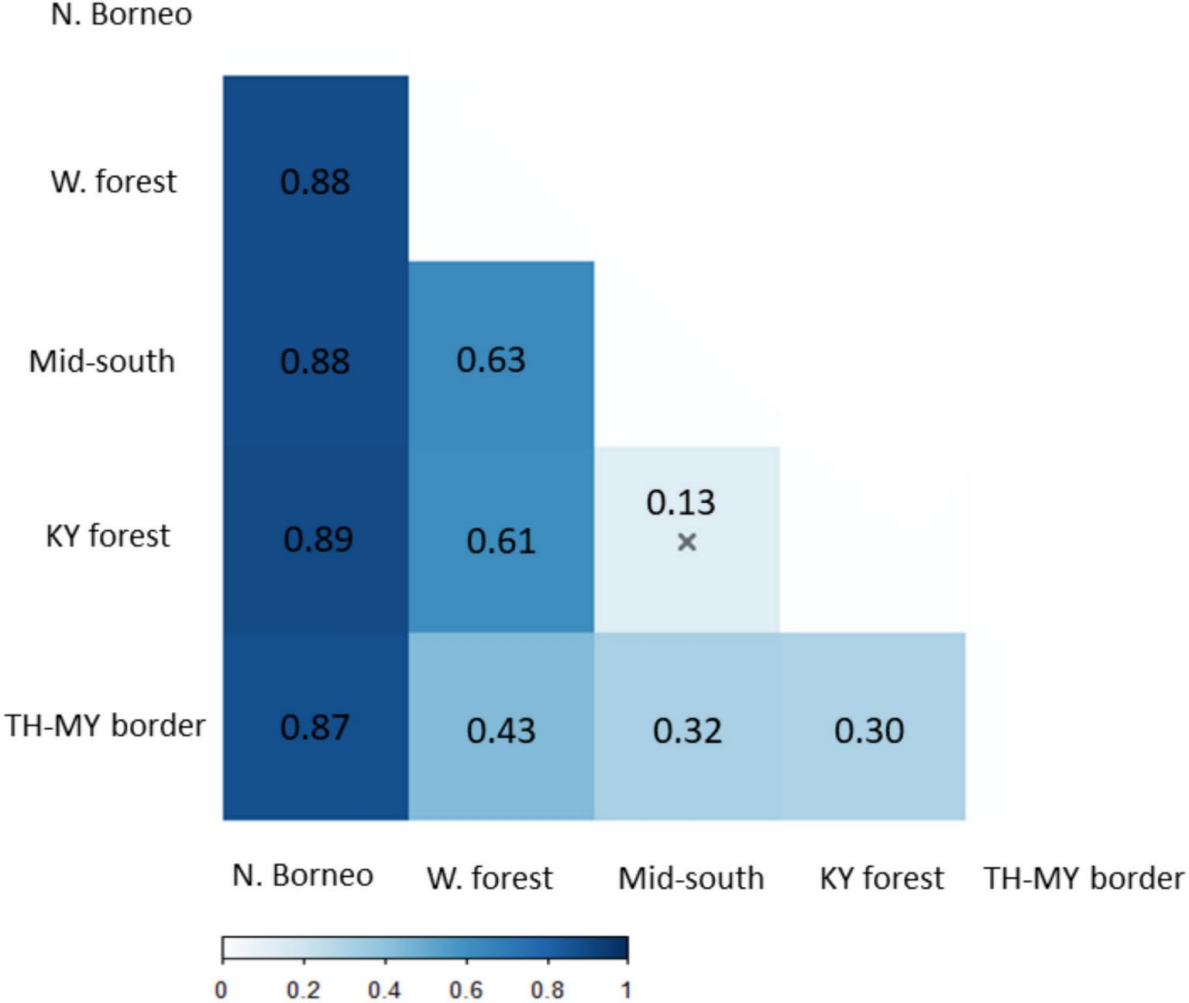
Genetic distance matrix based on *F*_ST_ among five geographic areas selected according to the sampling locations within discrete existing pangolin forest habitats. Pairwise *F*_ST_ values values are significant (*P* < 0.05), except the box marked ‘x’.

AMOVA analysis revealed significant genetic variation distributed among the five pangolin sampling localities (76% of variance, *P*-value < 0.01, Table.1), while the proportion of variance within populations was relatively low (23%). The pairwise *F*_ST_ matrix indicated significant differentiation of mtDNA haplotypes between all localities except one: the mid-south area and Khao Yai forest complex (*F*_ST_ = 0.13). The highest continental differentiation occurred between western forest complex and mid-south area (*F*_ST_ = 0.63), followed by the western forest complex and Khao Yai forest complex (*F*_ST_ = 0.61) (Fig. 4), supporting a historic east–west split within Thailand, visible in Fig. 3. One striking result, given the lack of shared haplotypes across the KPL, was the relatively low *F*_ST_ values for haplotype differentiation between the Thailand-Malaysia border region, and the western forest complex, Khao Yai forest complex and mid-South Thailand regions (pairwise *F*_ST_ = 0.30 to 0.43). Consistent with previous research, the northern Borneo population exhibited the greatest pairwise *F*_ST_ values, ranging from 0.87 to 0.89.

**Table 1:** Analysis of molecular variance (AMOVA) results with the six populations according to the phylogenetic tree and the haplotype network result.

Source of variation	Sum of squares	Variance components	Percentage variation	*P*-value
Among populations	5696.83	75.50	76.72	P < 0.01
Within populations	2177.01	22.91	23.28	
Total	7873.84	98.41

## Discussion

Our results provide the first detailed assessment of evolutionary genetic diversity in the Sunda pangolin throughout Thailand, and reveal multiple previously unknown mitochondrial DNA lineages. The most prominent pattern in our data was the clear phylogeographic structuring within continental Southeast Asia, where we observed five distinct clades with notable geographic distributions. Three clades were concentrated in the southern Thai-Malay border region (red, black and orange) and two (green and blue) were distributed further north in Thailand, stretching up to Myanmar and China, respectively. This north–south separation corresponds to the Kangar-Pattani Line (KPL), the biogeographical barrier which divides the Indochinese and Sundaic bioregions^46,47^. This division is thought to be driven by a climate-mediated shift in forest habitat type^48,49^. This result may explain the findings of a previous study^28^ that relied primarily on seized Sunda pangolins, but which postulated the separation of the species into two main continental groups: Yunnan / Myanmar and Malaysia, based on nuclear DNA data. The KPL has typically been observed to separate flora, in relation to climatic variation in Malaysia at 6°N to 7°N^46^, while the Isthmus of Kra (11°N to 13°N) is better known as a zoogeographic boundary^47^. However, our results indicate separation around the KPL, similar to other mammalian species such as the grey-bellied (*Callosciurus caniceps*) and Asian red-cheeked (*Dremomys rufigenis*) squirrels^50^, and invertebrates such as the fruit fly (*Zeugodacus cucurbitae*)^51^. The Sunda pangolin phylogeographic patterns we have inferred suggest a complex evolutionary history potentially involving multiple migration events across the KPL.

North of the KPL, the geographic distributions of the two main clades show increasing divergence, with an east–west split emerging and becoming more defined towards northern Indo-China, although further sampling in Myanmar, Lao PDR and Vietnam would be required to verify this pattern. The distribution of the western (green) clade, predominant in Thailand’s western forest complex and other western sampling localities in Thailand and Myanmar, relative to the eastern (blue) clade found in the Khao Yai forest complex, mid-south Thailand and eastern China, suggests a historical parapatric distribution of Sunda pangolin lineages. The haplotype network analysis revealed further structuring within these two clades, with individual haplotypes grouping largely according to geographic origin, reinforcing the phylogeographic distribution patterns of Sunda pangolin in Thailand.

Within central Thailand, genetic divergence of pangolin lineages between the western forest complex and the Khao Yai forest complex is analogous to the speciation pattern observed within and between other species. For example, Tickell’s brown hornbill (*Anorrhinus tickelli*) and Austen’s brown hornbill (*Anorrhinus austeni*), originally classified as conspecific subspecies, show a similar east–west partitioning, with *A. tickelli* occupying the western forest complex, while *A. austeni* is found in the Khao Yai forest complex^52^. Hybridization between white-handed (*Hylobates lar*) and pileated (*Hylobates pileatus*) gibbons in a contact zone in Khao Yai National Park reflects another east–west phylogenetic split among closely related taxa in this region^53^, indicating the possible presence of an east–west biogeographic division in Thailand, which could be explained by the Chao Phraya River Basin acting as a natural barrier between populations in some species, such as the gibbons and squirrel^54,55^.

In contrast, Sunda pangolin clades to the south of the KPL (red, orange, and black) were not observed to exhibit geographic structuring and appear to be distributed sympatrically around the Thai-Malay border. Within each clade, there was no apparent association between haplotype and sampling locality, while the positioning of samples within the orange and yellow clusters in the network emphasized the degree of mitochondrial genetic divergence in this region. The results of pairwise *F*_ST_ analysis for the Thai-Malay border region were curious, suggesting closer genetic affinity to both the western and Khao Yai forest complexes than these forest complexes had for each other, despite sharing some haplotypes. We interpret this as being due to the breadth of haplotype variation within the Thai-Malay border region (red, orange, black clusters) and the shorter network distances between these clusters and each of the western (green) and Khao Yai (blue) forest complex clusters, compared to the genetic divergence between the forest complexes. This pattern is particularly notable given that Sunda pangolin has relatively limited capacity for large-scale migration^56,57^.

The results also provide further evidence of the division between the two phylogenetic groups lineages previously recorded on Borneo^27^, whereby the pangolins in west/south Borneo appear to be associated with a mitogenome clade that includes pangolins from Peninsular Malaysia, Thailand, Myanmar, and China, rather than the neighbouring northern Borneo clade. In this study, the finding of a mitogenome haplotype in southern Thailand that is much more closely related to the west/south Borneo haplotype (with “PangSrwk”), compared to haplotypes previously observed, significantly strengthens the phylogenetic link between these geographic regions and reinforces the deep phylogenetic division within Borneo^27^. This pattern aligns with mitogenome phylogenies of shrews^58^, colugo^59^ and sun bears^60^, which also show shared haplotypes between western Borneo and the Southeast Asian continent.

Overall, the levels of genetic differentiation observed among the major Sunda pangolin mitogenome clades was generally lower than intraspecific divergences characterized in the Chinese pangolin^28^. Based on 782 bp of CYTB (the region utilised by^28^), the largest divergence within the Sunda pangolin, between the northern Borneo and other clades, were separated by 17 substitutions, while the two major Chinese pangolin clades were separated by 37 substitutions (Sup. Figure S6). Intraspecific diversity in the other two Asian pangolin species is not well-established.

### Conservation genetic management

The strong regional genetic differentiation and distribution of lineage diversity observed in this species has important implications for conservation management. The geographic patterns of mitochondrial DNA divergence on the continent, either side of the Kangar-Pattani Line, while strong, do not warrant designation as ESUs due to the lack of reciprocal monophyly. However, the high diversity of Sunda pangolin mtDNA haplotype clades observed in this relatively small area straddling northern peninsular Malaysia and southern Thailand (Sup. Figures S4 and Sup. Table S3), highlighted by the network analysis, identifies this region as an evolutionary genetic hotspot for the species. In terms of current conservation management, Malaysia—Thailand border area should be prioritised to preserve the species’ genetic diversity and evolutionary potential. Despite existing transboundary protected areas, increasing habitat fragmentation threatens these unique populations, highlighting the urgent need for enhanced connectivity between protected areas in both countries.

Within Thailand, the western forest complex and Khao Yai forest complex may also warrant separate Management Unit status, based on their high genetic differentiation (*F*_ST_ = 0.61) (but not entirely distinct) and the fact that these two habitats are separated by a large waterway (Chao Phraya River) and more recently by a large urban development north of Bangkok. As pangolins are known to swim and survive in peri-urban environments, neither factor necessarily comprises an absolute barrier to gene flow, and further research to characterise nuclear genetic differentiation is required; however, it seems likely that considering separate Sunda pangolin management units between eastern and western Thailand is appropriate.

The primary phylogeographic split separating the northern Borneo population from Sundaland and Indochina has been previously observed^27^, albeit with a slightly smaller dataset, and the current findings reinforce this distinction that supports the recognition of the north Borneo pangolin as a separate Evolutionary Significant Unit (ESU)^61,62^. Further sampling, analyses of nuclear DNA markers and evaluation of morphological traits in the northern Borneo are required to determine whether or not the northern Borneo pangolin population merits further taxonomic elevation, as has been suggested^27^.

### Implications for traceability and repatriation

Beyond informing regional in situ conservation management of the Sunda pangolin and its habitat, this study can support efforts to trace the geographic origin of seized pangolins or pangolin scales, as well as helping to reconstruct routes of illegal wildlife trade^63,64^. The discrete distribution of many haplotypes observed in this study may provide clues as to the geographic origin of the trafficked pangolins for forensic intelligence^65^. While this approach is disrupted by human-mediated animal translocation and limited by the broad geographic scale of certain haplotypes, mitochondrial DNA data has the advantage of being readily generated in most wildlife DNA forensic laboratories and readily shared among them, enabling traceability, for example in African elephants^66^ and lions^67^. Our study adds to several existing mitochondrial DNA studies on the geographic provenance of pangolins^25,27,28,68,69^, providing a broad scale geographic provenance information across an increasingly comprehensive range of species and distributions. For the Sunda pangolin, additional samples from Indonesia, in particular Sumatra and Java, identified as a major origin of pangolin trafficking between 2010–2015^70^, alongside Borneo, Vietnam, Lao PDR, and Cambodia, would enable a more complete characterization of genetic variation to identify the geographic origins across the species’ range. Given the relatively deep intra-specific phylogenetic divisions observed in this study, traceability is also important to ensure rescued or confiscated pangolins can be returned to appropriate locations that match their genetic provenance, to optimise their chance of survival and to maintain genetically cohesive populations and evolutionary processes in their native locations^71,72^.

## Conclusion

This study has provided the first conservation genetic management data on the Sunda pangolin in Thailand, revealing mitochondrial DNA diversity hotspots and phylogeographic structure that can be used to aid in species and habitat management and trade traceability. Further genetic studies that expand geo-referenced sample coverage and employ nuclear genomic markers will enhance our understanding of fine-scale population genetic processes.

## Supplementary Information

Supplementary Information.

## Data Availability

The sequences produced in this work can be found in the GenBank database with the accession numbers PP266608-PP266638.
