# Supplementary material for: Mitogenomic analysis of Thai Sunda pangolins reveals regional phylogeography and informs conservation management
Source: Sci Rep. 2025 Apr 23;15:14067. doi: 10.1038/s41598-025-97182-1 (PMC12018953; doi:10.1038/s41598-025-97182-1)
Supplement: Supplementary file 1 — Supplementary Information. [file 41598_2025_97182_MOESM1_ESM.docx]

**Title**

Mitogenomic analysis of Thai Sunda pangolins reveals regional phylogeography, and informs conservation management

**Authors:** Nattapong Banterng^1,2^, Kyle Ewart^3,4^, Frankie Thomas Sitam^5^ and Rob Ogden^1,4^

**Affiliations:**

1. Royal (Dick) School of Veterinary Studies and the Roslin Institute, University of Edinburgh, Easter Bush Campus, EH25 9RG, UK
2. Department of National Parks, Wildlife and Plant Conservation, Bangkok, Thailand
3. School of Life and Environmental Sciences, University of Sydney, Sydney, New South Wales, 2050, Australia
4. TRACE Wildlife Forensics Network, Edinburgh, EH12 6LE, UK
5. National Wildlife Forensic Laboratory (NWFL), Department of Wildlife and National Parks (DWNP/PERHILITAN), Kuala Lumpur, Malaysia

Email: N.Banterng@sms.ed.ac.uk

**Supplementary Materials**

**Table S1.** Details of Sunda pangolin sequenced in this study, along with Accession numbers, haplotype ID and references of sequence data obtained. The samples were derived from the Department of National Parks, Wildlife and Plant Conservation (DNP), Thailand.

| **Sample name** | **NCBI Accession No.** | **Haplotype ID** | **Geographic provenance** | **Collection** | **Sampling locality** | **Sample type** | **Study** | **Source** |
| --- | --- | --- | --- | --- | --- | --- | --- | --- |
| A2 | PP266608 | MJ_mt_01 | Western forest complex | DNP | Salakpra Wildlife Sanctuary | Blood | Current study | Rescued |
| A3 | PP266609 | MJ_mt_02 | Western forest complex | DNP | Salakpra Wildlife Sanctuary | Blood | Current study | Rescued |
| A4 | PP266610 | MJ_mt_03 | Western forest complex | DNP | Ladyao, Nakhon Sawan | Blood | Current study | Rescued |
| A5 | PP266611 | MJ_mt_04 | Western forest complex | DNP | Khaoson Wildlife breeding station | Tissue | Current study | Rescued |
| A7 | PP266612 | MJ_mt_05 | Western forest complex | DNP | Krasiao Dam, Suphan Buri | Blood | Current study | Rescued |
| B1 | PP266613 | MJ_mt_06 | Khao Yai forest complex | DNP | Saraburi | Blood | Current study | Rescued |
| B3 | PP266614 | MJ_mt_07 | Khao Yai forest complex | DNP | Tublan National Park | Blood | Current study | Rescued |
| B5 | PP266615 | MJ_mt_08 | Khao Yai forest complex | DNP | Panggae, Pakchong | Blood | Current study | Rescued |
| B7 | PP266616 | MJ_mt_09 | Khao Yai forest complex | DNP | Na Di, Prachin Buri | Blood | Current study | Rescued |
| B8 | PP266617 | MJ_mt_10 | Khao Yai forest complex | DNP | Tublan National Park | Blood | Current study | Rescued |
| B9 | PP266618 | MJ_mt_11 | Khao Yai forest complex | DNP | Nong Prue, Bang Lamung | Blood | Current study | Rescued |
| B10 | PP266619 | MJ_mt_12 | Khao Yai forest complex | DNP | Khao Yai National Park | Blood | Current study | Rescued |
| B11 | PP266620 | MJ_mt_13 | Khao Yai forest complex | DNP | Si Khio,Nakhon Ratchasima | Blood | Current study | Rescued |
| B12 | PP266621 | MJ_mt_14 | Khao Yai forest complex | DNP | Khok Pee Khong, Sa Kaeo | Blood | Current study | Rescued |
| B13 | PP266622 | MJ_mt_15 | Khao Yai forest complex | DNP | Klonghad, Sa Kaeo | Blood | Current study | Rescued |
| B14 | PP266623 | MJ_mt_09 | Khao Yai forest complex | DNP | Krabin Buri, Prachin Buri | Blood | Current study | Rescued |
| C1 | PP266624 | MJ_mt_17 | Mid-south Thailand | DNP | Khirirat Nikhom, Surat Thani | Blood | Current study | Rescued |
| C2 | PP266625 | MJ_mt_18 | Mid-south Thailand | DNP | Makham Tia,Surat Thani | Blood | Current study | Rescued |
| C5 | PP266626 | MJ_mt_19 | Mid-south Thailand | DNP | Thamsing, Chumphon | Blood | Current study | Rescued |
| C6 | PP266627 | MJ_mt_20 | Mid-south Thailand | DNP | Ban Na, Chumphon | Tissue | Current study | Rescued |
| C9 | PP266628 | MJ_mt_21 | Mid-south Thailand | DNP | Langsuan, Chumphon | Tissue (Hair) | Current study | Rescued |
| X11 | PP266638 | MJ_mt_52 | Mid-south Thailand | DNP | Talumphuk, Nakhon Si Thammarat | Blood | Current study | Rescued |
| D1 | PP266629 | MJ_mt_23 | Far south Thailand | DNP | Ton Nga Chang Wildlife Sanctuary | Blood | Current study | Rescued |
| D2 | PP266630 | MJ_mt_24 | Far south Thailand | DNP | Buketa, Narathiwat | Tissue | Current study | Rescued |
| D3 | PP266631 | MJ_mt_25 | Far south Thailand | DNP | Khok Khian, Narathiwat | Tissue | Current study | Rescued |
| D5 | PP266632 | MJ_mt_26 | Far south Thailand | DNP | Hala Bala Wildlife Sanctuary | Tissue | Current study | Confiscated |
| D7 | PP266633 | MJ_mt_27 | Far south Thailand | DNP | Khok Khian, Narathiwat | Blood | Current study | Rescued |
| X6 | PP266636 | MJ_mt_50 | Far south Thailand | DNP | Palian, Trang | Blood | Current study | Rescued |
| X3 | PP266634 | MJ_mt_48 | Central Thailand | DNP | Kaeng Sopha, Phitsanulok | Blood | Current study | Rescued |
| X5 | PP266635 | MJ_mt_49 | Northern Thailand | DNP | Chiang Dao, Wildlife Sanctuary | Blood | Current study | Rescued |
| X10 | PP266637 | MJ_mt_51 | Northern Thailand | DNP | Huai Phueng Wang Yao Non-Hunting Area | Blood | Current study | Rescued |
| PangSrwk | OR327007 | MJ_mt_43 | West Borneo | GenBank | Padawan, Kuching, Sarawak | Tissue | Sitam et al., 2023 | - |
| MJ565  (Offspring of MJ566) | OR327008 | MJ_mt_40 | ­ Peninsular Malaysia | GenBank | PKHL Sungkai, Perak, Peninsular Malaysia (offspring of MJ566) | Tissue | Sitam et al., 2023 | - |
| MJ566 | OR327009 | MJ_mt_40 | Peninsular Malaysia | GenBank | Kg. Jernang, Sungkai, Perak, Peninsular Malaysia | Tissue | Sitam et al., 2023 | - |
| MJ562 | OR327010 | MJ_mt_39 | ­ Peninsular Malaysia | GenBank | Teluk Cempedak, Kuantan, Pahang, Peninsular Malaysia | Tissue | Sitam et al., 2023 | - |
| MJ553 | OR327011 | MJ_mt_32 | ­ Peninsular Malaysia | GenBank | Kuantan, Pahang, Peninsular Malaysia | Hair | Sitam et al., 2023 | - |
| MJ564 | OR327012 | MJ_mt_32 | Peninsular Malaysia ­ | GenBank | Lenggong, Perak, Peninsular Malaysia | Tissue | Sitam et al., 2023 | - |
| MJ556 | OR327013 | MJ_mt_34 | Peninsular Malaysia | GenBank | Pengkalan Hulu, Perak, Peninsular Malaysia | Tissue | Sitam et al., 2023 | - |
| MJ560 | OR327014 | MJ_mt_37 | ­ Peninsular Malaysia | GenBank | Gerik, Perak, Peninsular Malaysia | Blood | Sitam et al., 2023 | - |
| MJ555 | OR327015 | MJ_mt_33 | ­ Peninsular Malaysia | GenBank | Pengkalan Hulu, Perak, Peninsular Malaysia | Tissue | Sitam et al., 2023 | - |
| MJ557 | OR327016 | MJ_mt_33 | ­ Peninsular Malaysia | GenBank | Pengkalan Hulu, Perak, Peninsular Malaysia | Tissue | Sitam et al., 2023 | - |
| MJ558 | OR327017 | MJ_mt_35 | ­ Peninsular Malaysia | GenBank | Lahat, Ipoh, Perak, Peninsular Malaysia | Blood | Sitam et al., 2023 | - |
| MJ561 | OR327018 | MJ_mt_38 | ­ Peninsular Malaysia | GenBank | Bidor, Perak, Peninsular Malaysia | Blood | Sitam et al., 2023 | - |
| MJ552 | OR327019 | MJ_mt_31 | ­ Peninsular Malaysia | GenBank | Raub, Pahang, Peninsular Malaysia | Tissue | Sitam et al., 2023 | - |
| MJ559 | OR327020 | MJ_mt_36 | ­ Peninsular Malaysia | GenBank | Kota Bharu, Kelantan, Peninsular Malaysia | Blood | Sitam et al., 2023 | - |
| MJ567 | OR327021 | MJ_mt_41 | ­ Peninsular Malaysia | GenBank | PKHL Sungkai, Perak, Peninsular Malaysia | Tissue | Sitam et al., 2023 | - |
| C10 | OR327022 | MJ_mt_22 | ­ North Borneo | GenBank | Kota Kinabalu, Sabah | Hair | Sitam et al., 2023 | - |
| MJ551 | OR327023 | MJ_mt_30 | North Borneo | GenBank | Labuan, Sabah | Tissue | Sitam et al., 2023 | - |
| G01 | OR327024 | MJ_mt_28 | North Borneo | GenBank | Ranau, Sabah | Blood | Sitam et al., 2023 | - |
| P05 | OR327025 | MJ_mt_42 | North Borneo | GenBank | Sandakan, Sabah | Blood | Sitam et al., 2023 | - |
| B01a | OR327026 | MJ_mt_16 | North Borneo | GenBank | Lower Kinabatangan Wildlife Sanctuary, Sabah | Blood | Sitam et al., 2023 | - |
| S01 | OR327027 | MJ_mt_44 | North Borneo | GenBank | Plantation near Kampung Paris, Kinabatangam, Sabah | Blood | Sitam et al., 2023 | - |
| TUAH | OR327028 | MJ_mt_44 | North Borneo | GenBank | Sandakan, Sabah | Hair | Sitam et al., 2023 | - |
| SRR9018633 | SRR9018633 | MJ_mt_45 | ­- | GenBank | Kachin, Myanmar | - | Hu et al., 2020 | - |
| SRR9018664 | SRR9018664 | MJ_mt_46 | ­- | GenBank | Yunnan Province, China | - | Hu et al., 2020 | - |
| SRR9018665 | SRR9018665 | MJ_mt_47 | ­- | GenBank | Yunnan Province, China | - | Hu et al., 2020 | - |
| MG196309 | MG196309 | MJ_mt_29 | ­- | GenBank | Guangxi, China | Tissue | Gaubert et al., 2018 | - |
| MG196302 | MG196302 | MJ_mt_19 | ­ Mid-south Thailand | GenBank | Kapoe, Thailand | Tissue | Gaubert et al., 2018 | - |
| MG196308 | MG196308 | ­- | ­- | GenBank | *Manis culionensis* | - | Gaubert et al., 2018 | - |
| SRR25256520 | SRR25256520 | ­- | ­- | GenBank | *Manis mysteria* | - | Gu et al., 2023 | - |
| SRR25256582 | SRR25256582 | ­- | ­- | GenBank | *Manis mysteria* | - | Gu et al., 2023 | - |
| MG196305 | MG196305 | ­- | - | GenBank | *Manis crassicaudata* | - | Gaubert et al., 2018 | - |
| MG196307 | MG196307 | ­- | - | GenBank | *Manis pentadactyla* | - | Gaubert et al., 2018 | - |

**Table S2.** Haplotype statistics for the mitogenome (15,408 bp), separated into groups based on coloured clades observed in the phylogenetic tree (Figure 1.)

| **Group** | **Nucleotide diversity** | **No. of samples** | **No. of haplotypes** | **Haplotype diversity** |
| --- | --- | --- | --- | --- |
| Blue | 0.00153 | 18 | 16 | 0.9869 |
| Black | 0.00122 | 8 | 7 | 0.9643 |
| Red | 0.00185 | 8 | 7 | 0.9643 |
| Green | 0.00164 | 12 | 12 | 1 |
| Purple | 0.00529 | 7 | 6 | 0.9524 |
| Orange | 0.00143 | 3 | 2 | 0.6667 |
| Yellow | 0.00409 | 2 | 2 | 1 |

**Table S3.** The haplotype statistics using the mitogenome (15,408 bp), separated into groups based on geographical.

| **Group** | **Nucleotide diversity** | **No. of samples** | **No. of haplotypes** | **Haplotype diversity** |
| --- | --- | --- | --- | --- |
| TH-MY border | 0.00346 | 20 | 17 | 0.9842 |
| Northern Borneo | 0.00529 | 7 | 6 | 0.9524 |
| Mid-South | 0.00230 | 6 | 6 | 1 |
| West | 0.00138 | 5 | 5 | 1 |
| KY | 0.00212 | 11 | 10 | 0.9818 |

**Table S4.** The 14 partitions with their respective substitution models, resulting from PartitionFinder2.

| **Gene** | **Partition** | **Model** |
| --- | --- | --- |
| tRNA-Phe, tRNA-Leu1, tRNA-Gly | 1 | TIM+G |
| 12S-Rrna, 16S-rRNA, tRNA-Ile, tRNA-Ile, tRNA-Trp | 2 | TVM+I+G |
| tRNA-Val | 3 | TRN |
| ND1, ATP6, ND4, tRNA-Glu, Cytb | 4 | GTR+I |
| tRNA-Gln, tRNA-Ser, tRNA-Asp, ND3, ND4L | 5 | TIM+G |
| tRNA-Met, tRNA-Pro | 6 | HKY+I+G |
| ND2, ATP8 | 7 | TRN+G |
| tRNA-Ala, tRNA-Thr, D-loop | 8 | HKY+I+G |
| tRNA-Asn, tRNA-Cys, tRNA-Tyr, COX1, COX2, COX3 | 9 | GTR+I |
| tRNA-Lys | 10 | TRN+G |
| tRNA-Arg, tRNA-His | 11 | HKY |
| tRNA-Ser | 12 | TRN |
| tRNA-Leu2, ND6 | 13 | TRN+I+G |
| ND5 | 14 | TRN+I |


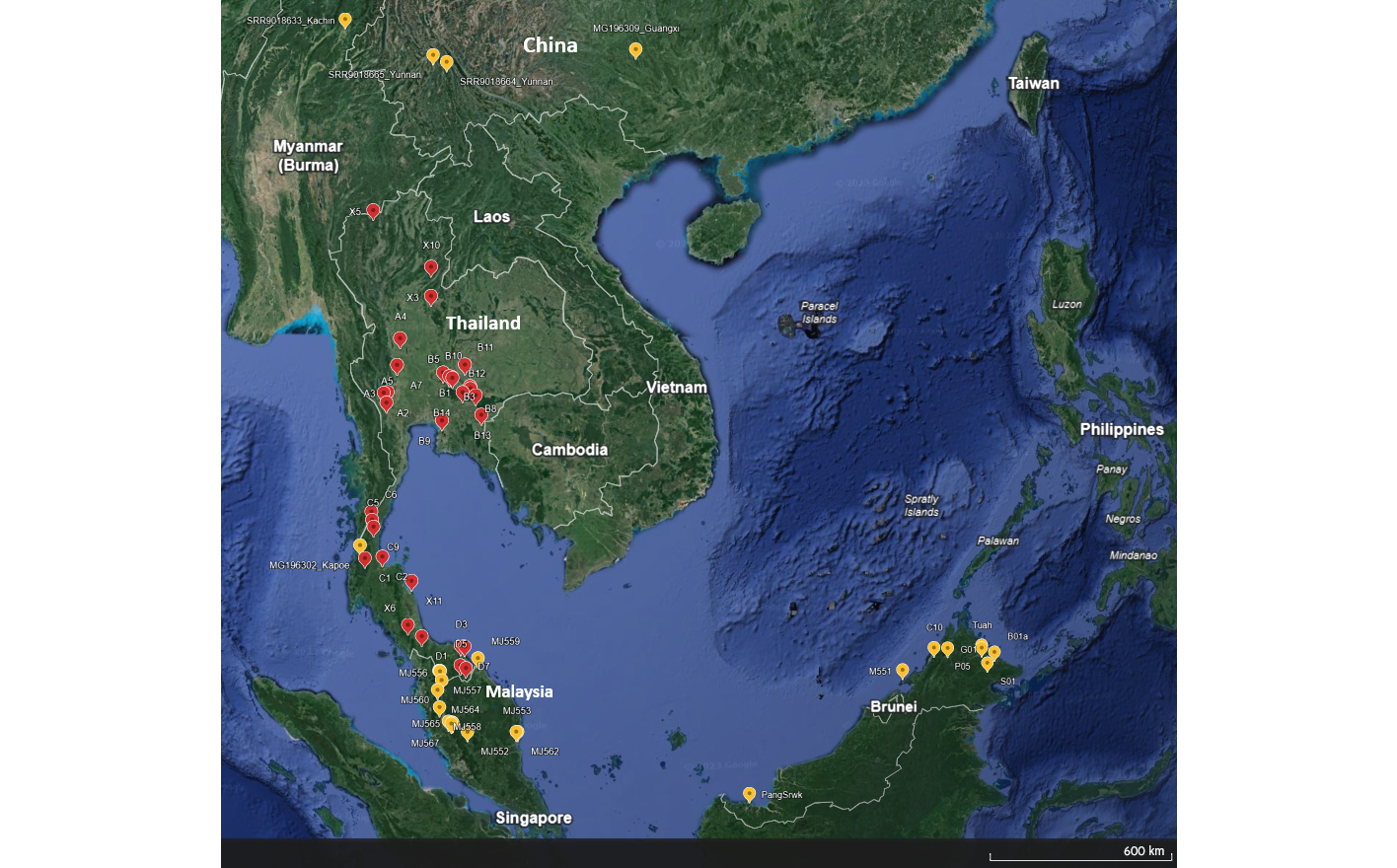


**Figure S1**. Map showing the origin of the 58 Sunda pangolin samples from Thailand, Peninsular Malaysia, Bornean Malaysia, China, and Myanmar used in this study. Red represents samples collected in this study, and yellow represents data from previous studies downloaded from NCBI GenBank. If specific coordinates were unavailable, locations are based on the region where samples were collected. The map created using Google Earth, v. 10.69.0.1 (https://earth.google.com/).


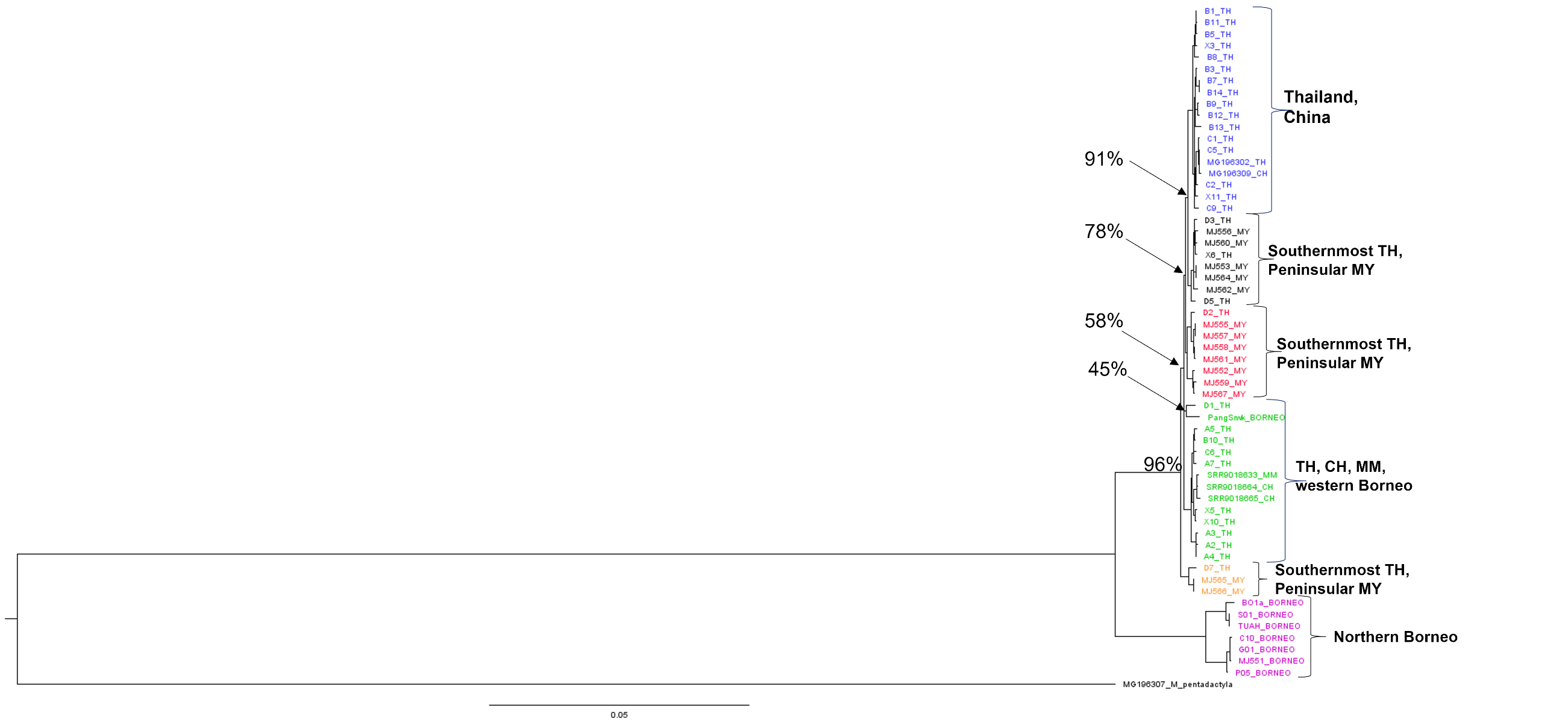


**Figure S2**. Maximum likelihood phylogenetic tree comprising 58 samples and whole mtDNA genomes (16,000 bp), implementing 1,000 bootstraps. Bootstrap support (%) is presented on key nodes. MG196307 (*Manis pentadactyla*) was used as an outgroup. Separated as colour-coded clades, TH represents samples from Thailand; MY from Peninsular Malaysia; CH from China; and MM from Myanmar.


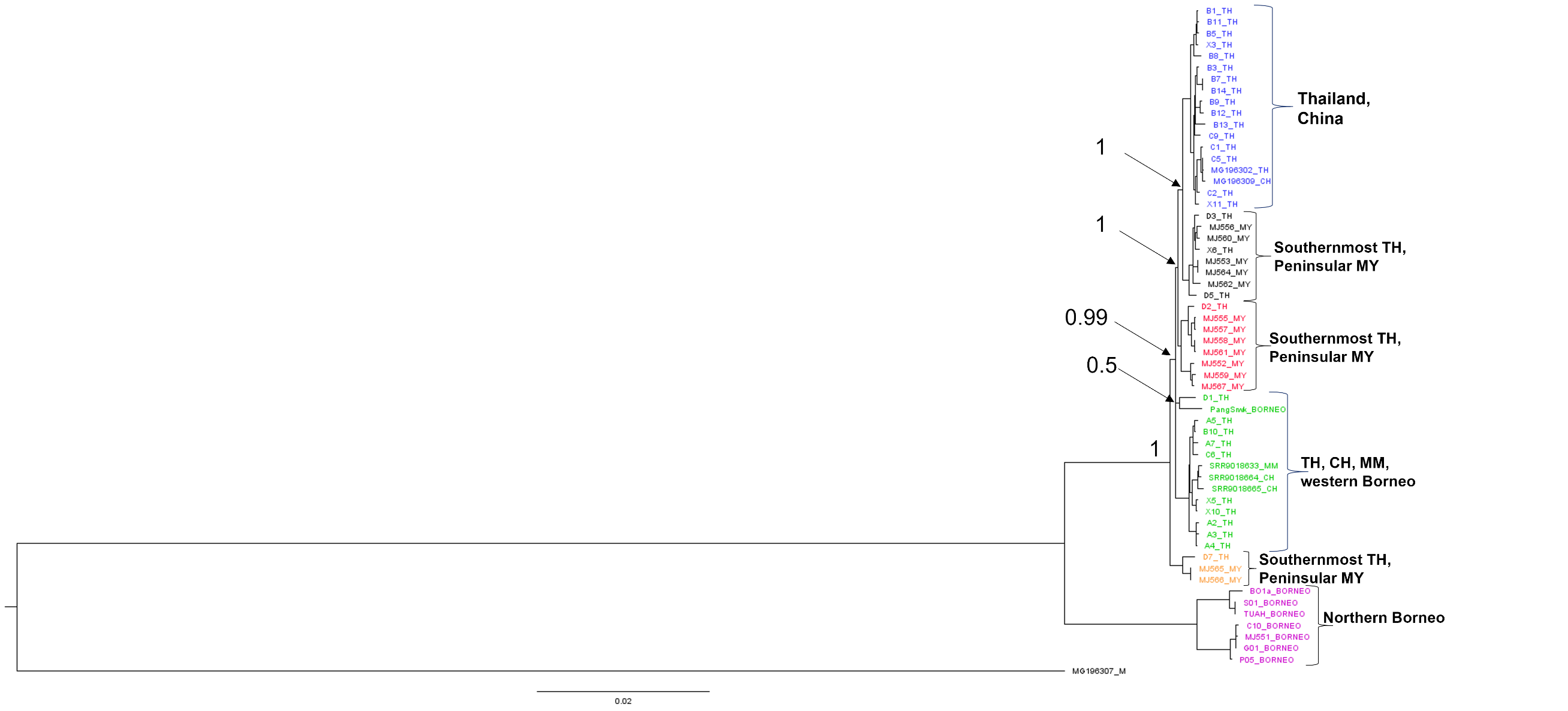


**Figure S3**. Bayesian phylogenetic tree comprising 58 samples and whole mtDNA genomes (16,000 bp). Posterior probabilities are presented on key nodes. Sequence MG196307 (*Manis pentadactyla*) was used as an outgroup. Distinct clades are colour-coded. TH = Thailand; MY = Peninsular Malaysia; CH - China; and MM = Myanmar.


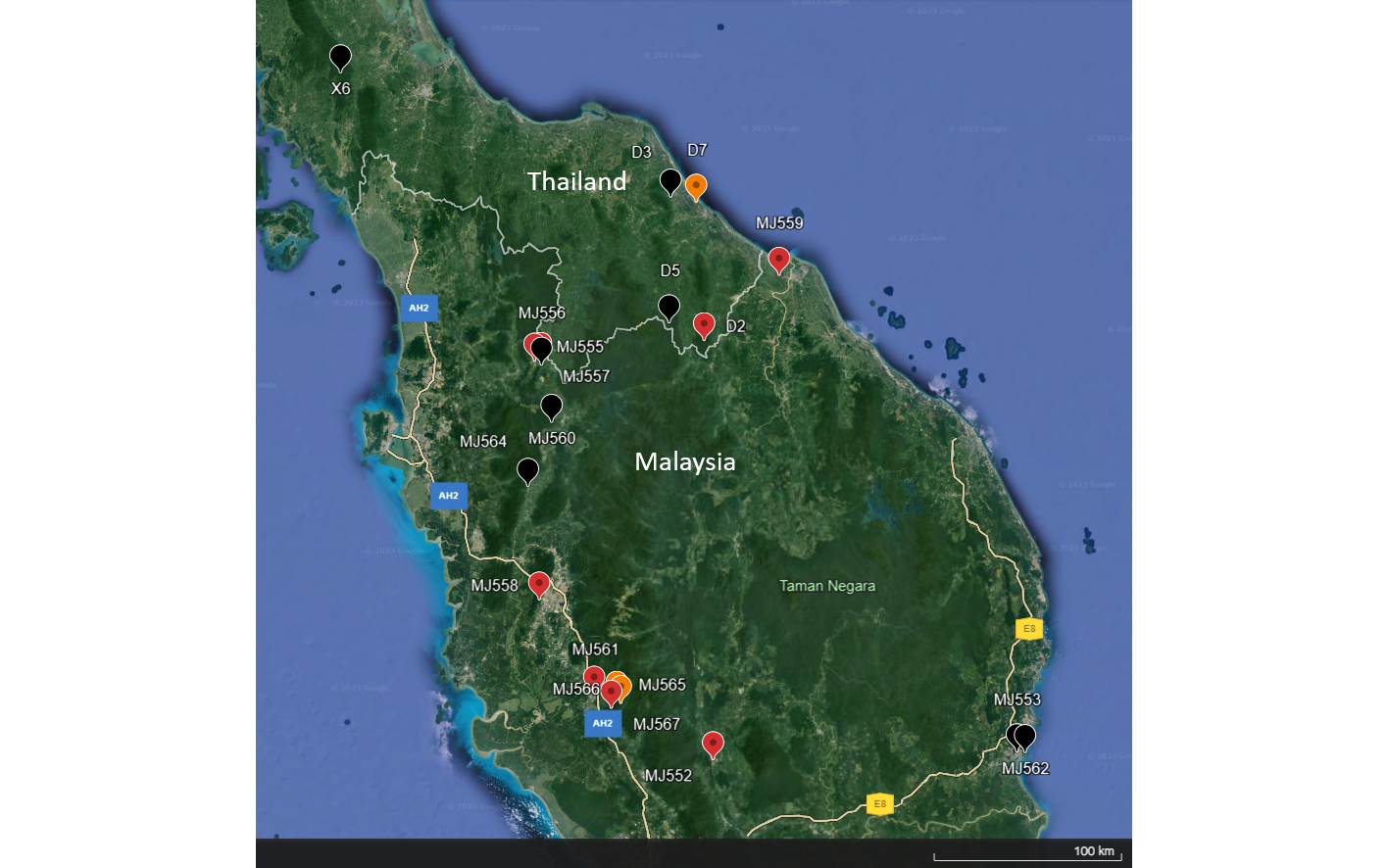


**Figure S4**. The geographic distribution of Sunda pangolin samples belonging to black, red and orange clades occurring in southernmost Thailand and Peninsular Malaysia (Thai – Malaysian border). The map created using Google Earth, v. 10.69.0.1 (https://earth.google.com/).

**
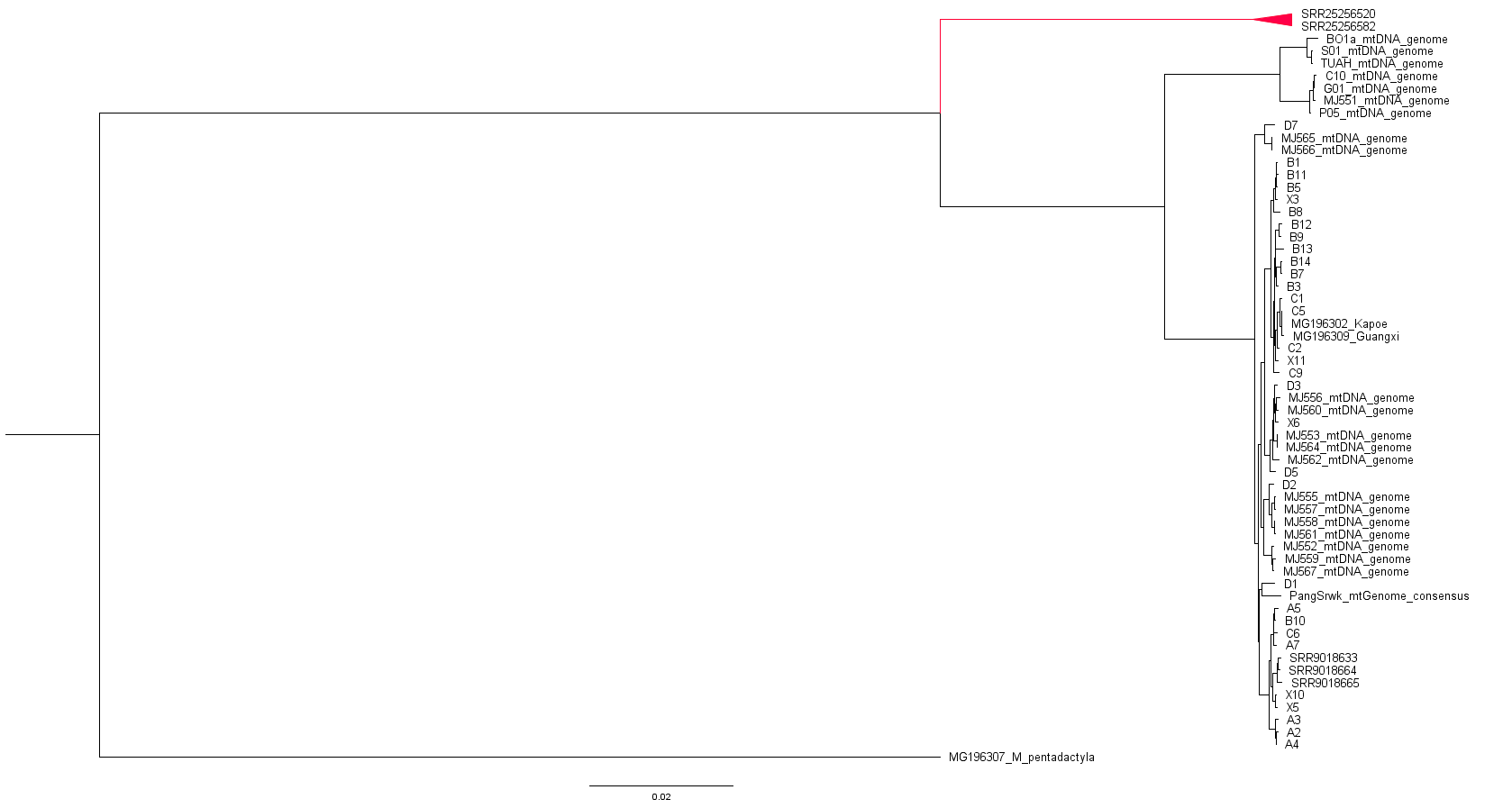
**

**Figure S5**. Bayesian phylogenetic tree of Sunda pangolin in this study with *Manis pentadactyla* and the recently proposed new species, *Manis mysteria* [1], highlighted in red, based on mitogenome sequence data (16,000 bp).


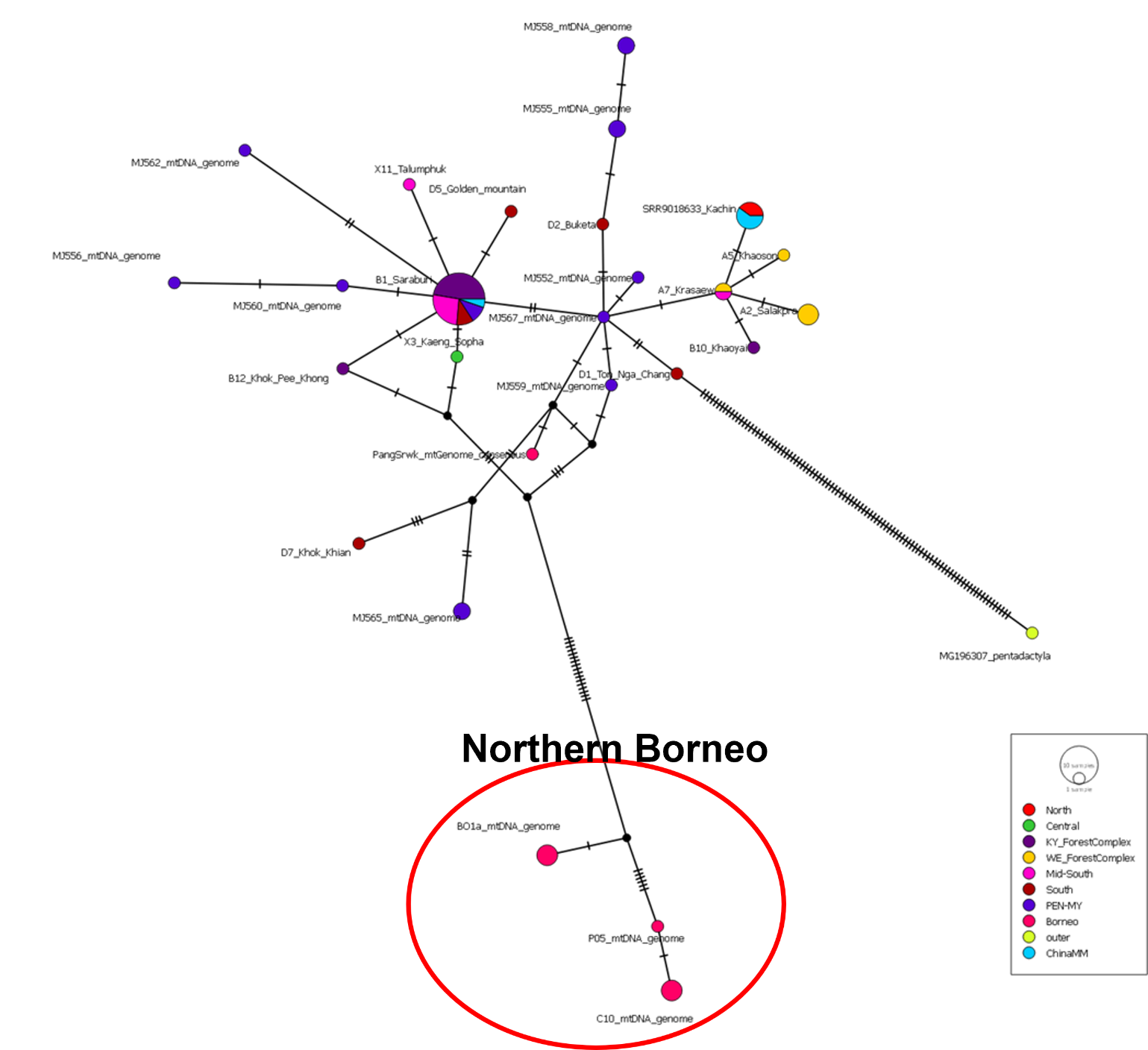


**Figure S6**. Haplotype network based on CYTB (782 bp) of the samples from Thailand, Peninsular Malaysia, Borneo, China, and Myanmar using the Median-Joining method in PopART v.1.7[2] according to Hu *et al*. (2020). The northern Borneo samples, all are framed in a red circle, showed the intraspecific divergences to other samples. The diameter of haplotype circle reflects the total number of samples, the small black circle represents the missing haplotype and hatch-marks across lines represent mutational steps between haplotypes.


1. Gu T-T, Wu H, Yang F, Gaubert P, Heighton SP, Fu Y, et al. Genomic analysis reveals a cryptic pangolin species. Proceedings of the National Academy of Sciences. 2023;120(40):e2304096120.

2. Leigh JW, Bryant D. POPART: full‐feature software for haplotype network construction. Methods in ecology and evolution. 2015;6(9):1110-6.
